# Effect of Intermittent Fasting Diet on Glucose and Lipid Metabolism and Insulin Resistance in Patients with Impaired Glucose and Lipid Metabolism: A Systematic Review and Meta-Analysis

**DOI:** 10.1155/2022/6999907

**Published:** 2022-03-24

**Authors:** Xiaojie Yuan, Jiping Wang, Shuo Yang, Mei Gao, Lingxia Cao, Xumei Li, Dongxu Hong, Suyan Tian, Chenglin Sun

**Affiliations:** ^1^Department of Clinical Nutrition, First Hospital of Jilin University, 1 Xinmin Street, Changchun, 130021 Jilin, China; ^2^Department of Endocrinology and Metabolism, First Hospital of Jilin University, 1 Xinmin Street, Changchun, 130021 Jilin, China; ^3^Division of Clinical Research, First Hospital of Jilin University, 1 Xinmin Street, Changchun, 130021 Jilin, China

## Abstract

The question of whether or not intermittent fasting diets improve the clinical indicators of glycolipid metabolism remains unclear. This study systematically reviewed the relevant clinical trials to evaluate the effects of intermittent fasting diet on glucose and lipid metabolism and insulin sensitivity in patients with metabolic syndrome. To evaluate the effect of intermittent fasting diet intervention on patients with disorders of glucose and lipid metabolism, random-effect or fixed-effect meta-analysis models were used to calculate the average difference before and after intermittent fasting diet intervention and the corresponding 95% confidence intervals (CIs). After intermittent fasting diet intervention, in terms of glucose metabolism, fasting blood glucose reduced by 0.15 mmol/L (95% CI: −0.23; −0.06), glycosylated hemoglobin reduced by 0.08 (95% CIs: −0.25; −0.10), insulin plasma levels reduced by 13.25 uUI (95% CIs: −16.69; −9.82), and HOMA-IR decreased by 0.31 on an average (95% CIs: −0.44; −0.19). In addition, BMI decreased by 0.8 kg/m^2^ (95% CIs: −1.32; −0.28), body weight reduced by 1.87 kg (95% CIs: −2.67; −1.07), and the waist circumference decreased by 2.08 cm (95% CIs: −3.06; −1.10). Analysis of lipid metabolism showed that intermittent fasting diet intervention effectively reduced the total cholesterol level by 0.32 mmol/L (95% CIs: −0.60; −0.05), low-density lipoprotein level by 0.22 mmol/L (95% CIs: −0.37; −0.07), and triglyceride level by 0.04 mmol/L (95% CIs: −0.15; −0.07). Intermittent fasting diets have certain therapeutic effects on blood glucose and lipids in patients with metabolic syndrome and significantly improve insulin resistance. It may be considered as an auxiliary treatment to prevent the occurrence and development of chronic diseases.

## 1. Introduction

Chronic diseases such as hyperglycemia, dyslipidemia, hypertension, and other diseases seriously affect the health condition. The abovementioned factors collectively contributed to metabolic syndrome (MS), which increases the risk of atherosclerotic diseases, cardiovascular disease, insulin resistance, and diabetes, as well as vascular and neurological complications (such as cerebrovascular accidents) [1–3]. In the United States, it was estimated that the prevalence of MS was 34.7% by the year 2016 [4]. The common pathological management of glucose and lipid metabolism is based on weight reduction, especially insulin resistance and hyperinsulinemia caused by central obesity. Insensitivity and resistance to insulin are also important pathogenic mechanisms of type 2 diabetes [5]. Effective dietary interventions are a preventive measure that can promote weight loss, improve glucose and lipid metabolism, improve insulin resistance, and prevent the occurrence and development of diabetes and cardiovascular diseases [6].

Intermittent fasting (IF) has been proposed as an alternative to restricted calorie diets as a means of weight control [7]. The IF diet includes various fasting patterns, such as alternate-day fasting (consuming no calories on fasting days), alternate day-modified fasting (consuming less than 25% of caloric requirements on fasting days), time-restricted fasting (restricting food intake at specific times of the day), and periodic fasting (fasting on one to two days per week). A retrospective study [8] found that IF diets produce gradual weight loss, lower blood pressure, have anticarcinogenic effects, and may even extend the lifespan. IF diets are not simply about enduring starvation, they represent a healthier lifestyle choice. A recent meta-analysis [7] reviewed the clinical studies of IF diets and found that they reduce adult body mass index (BMI) and insulin resistance. To the best of our knowledge, however, there have been no meta-analyses targeting clinical studies of the effects of IF diets on impaired glucose and lipid metabolism. IF diets have produced conflicting results in clinical studies of patients with impaired glucose and lipid metabolism. In support of IF, Sutton et al. [9] found that 5 weeks on an IF diet significantly improved the insulin sensitivity and blood pressure of male participants with impaired glucose and lipid metabolism. Furmli et al. [10] found that IF reversed and/or reduced insulin resistance while significantly reducing body weight, waist circumference, and HbA1c [10]. It also contributed to the maintenance of stable blood glucose levels after discontinuation of insulin use. In contrast, Alghamdi et al. [11] studied the effects of IF during Ramadan on glucose metabolism in patients with MS and found that HbA1c increased by 0.11% after a month. Harvie et al. [12, 13] found that 3 months on an IF diet significantly reduced the total cholesterol, triglyceride (TG), and low-density lipoprotein (LDL) levels of patients with impaired glucose and lipid metabolism. However, Sutton et al. [9] reported contradictory results, with increases in total cholesterol and TG in this demographic after 2 months of IF.

In the present study, we aimed to summarize clinical trials on the effects of IF diet intervention on glucose and lipid metabolism of patients with MS-impaired glucose and lipid metabolism in recent years worldwide, thereby exploring whether or not IF diets improve the clinical indicators of glycolipid metabolism. The current meta-analysis evaluated the actual efficacy of the IF diet using objective data and reported the effects of the IF diet on blood glucose, HbA1c and whether or not this intervention improves lipid metabolism and insulin sensitivity.

## 2. Methods and Materials

### 2.1. Literature Search

**Figure 1:**
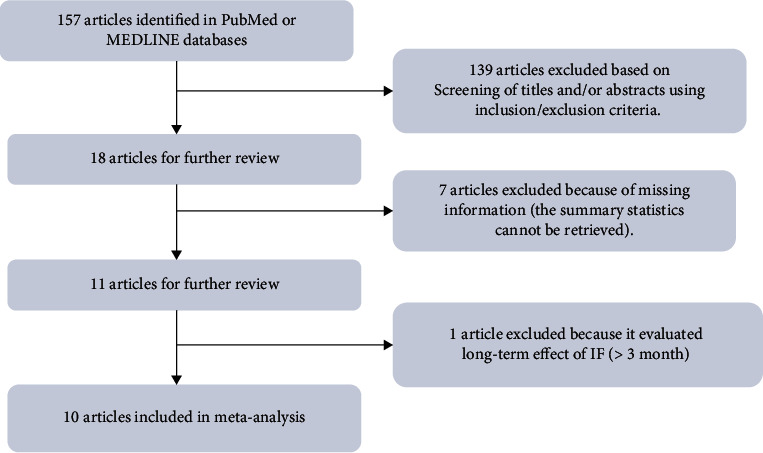
PRISMA diagram for the systematic review.

Only previous publications written in English were considered. A literature search was performed in the PubMed and MEDLINE databases by using the following keywords: intermittent fasting diet, obesity/overweight, randomized controlled trial, MS, and human. The end date of this literature search was June 5th, 2020. This meta-analysis was planned and performed following the Preferred Reporting Items for Systematic Reviews and Meta-Analysis Guideline (Figure 1). MS was defined as the presence of any of the following metabolic dysfunctions: obesity, hyperglycemia, dyslipidemia, or hypertension. Overweight was defined as BMI >25 kg/m^2^, and obesity was defined as BMI >30 kg/m^2^ according to the World Health Organization standard.

### 2.2. Inclusion/Exclusion Criteria

Inclusion criteria included the following: (1) the therapeutic dietary under consideration is IF; (2) the study was carried out on humans; and (3) the summary statistics of the mean difference between before and during the IF intervention is available (if the means before and during the IF diet is available, then the difference was taken to obtain the desired mean difference), along with their corresponding standard error or 95% confidence intervals (CIs) or *p* values. Based on either of the last two statistics, the corresponding statistics (i.e., the corresponding standard deviation of the mean difference) can be calculated. Given that many included studies are longitudinal, several time points may be retrieved in a single study.

Exclusion criteria included the following: (1) case reports, (2) meta-analyses or reviews, (3) studies of normal weight or lean subjects, (4) inadequate information to retrieve (or calculate) the necessary summary statistics, (5) data covering periods longer than 3 months, and (6) reviews, letters, conference abstracts, editorials, and commentaries.

Three of the authors (X.Y, J.W, and S.Y) independently reviewed the titles, abstracts, and full-text studies to determine eligibility for inclusion. Any disagreements were addressed by discussion or consultation with Dr. Sun and Dr. Tian.

### 2.3. Statistical Analysis

The effects of IF on MS were estimated by the mean difference before and after IF implementation and their corresponding 95% CIs in random-effect or fixed-effect meta-analysis models, which were determined through the heterogeneity of studies. Heterogeneity among studies was evaluated by Cochrane's *Q* statistic and the *I*^2^ statistical methods. If the corresponding *p* value was <0.05 and *I*^2^ > 0.5, a random-effect meta-analysis model was used. Otherwise, a fixed-effect meta-analysis model was chosen. To evaluate the possibility of potential bias, funnel plots in which effect sizes versus standard errors were diagramed and were made for each outcome and visually inspected. We further assessed for publication bias by using Egger's regression tests. All statistical analyses were carried out in the R software, version 3.5 (https://www.r-project.org).

## 3. Results

In total, we identified 157 potentially relevant citations from the PubMed and MEDLINE databases. After screening the titles and/or abstracts using our inclusion and exclusion criteria, 139 articles were excluded. The full text of 18 articles was evaluated. Of these, seven were excluded because of missing information (the summary statistics could not be retrieved), and one was excluded because of the study duration, as it was an evaluation of the long-term effects of IF. The remaining 10 articles fulfilled our criteria and were included in our meta-analysis. These comprised 10 randomized controlled trials with 12 types of intervention [9, 11–19]. Our search and screening process is summarized in Figure 1. Details such as the IF diet type and follow-up duration are summarized in Table 1. The effects of the IF diet on glucose metabolism, insulin resistance, weight loss, lipid metabolism, and blood pressure control of patients with impaired glucose and lipid metabolism were evaluated.

The effects of IF diets were systemically reviewed by within-subject comparisons of pre- and post-intervention measurements of relevant biomarkers. The variables measured to determine glucose metabolism included fasting glucose, insulin, HbA1c, and HOMA-IR levels, whereas TG, high-density lipoprotein (HDL), and LDL levels were used to assess lipid metabolism. Weight loss was measured using body weight, BMI, and waist circumference. In the meta-analysis, fixed-effect models were fit for BMI, waist circumference, SBP, HDL-C, HOMA-IR, and insulin, whereas random-effects models were adopted for other parameters according to their Cochrane's *Q* and *I*^2^ statistics.

**Figure 2:**
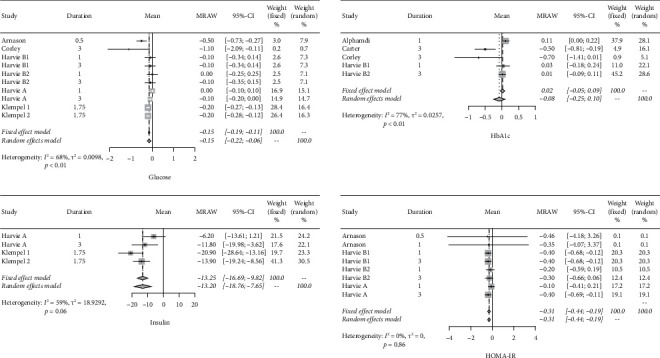
Forest plots for blood glucose, insulin, HbA1c, and HOMA-IR.

The fasting blood glucose level decreased by 0.15 mmol/L (95% CIs: −0.19; −0.11) after the IF diet. Concerning glycosylated hemoglobin, no significant change in HbAlc was found after the IF intervention even though the mean difference reduced by 0.08%. Insulin level decreased by 13.25 mU/L (95% CIs: -16.69; −9.82) on average after the IF intervention, whereas HOMA-IR decreased by 0.31 (95% CIs: −0.44; −0.19). The forest plots for these two carbohydrate metabolism indices are shown in Figure 2.

**Figure 3:**
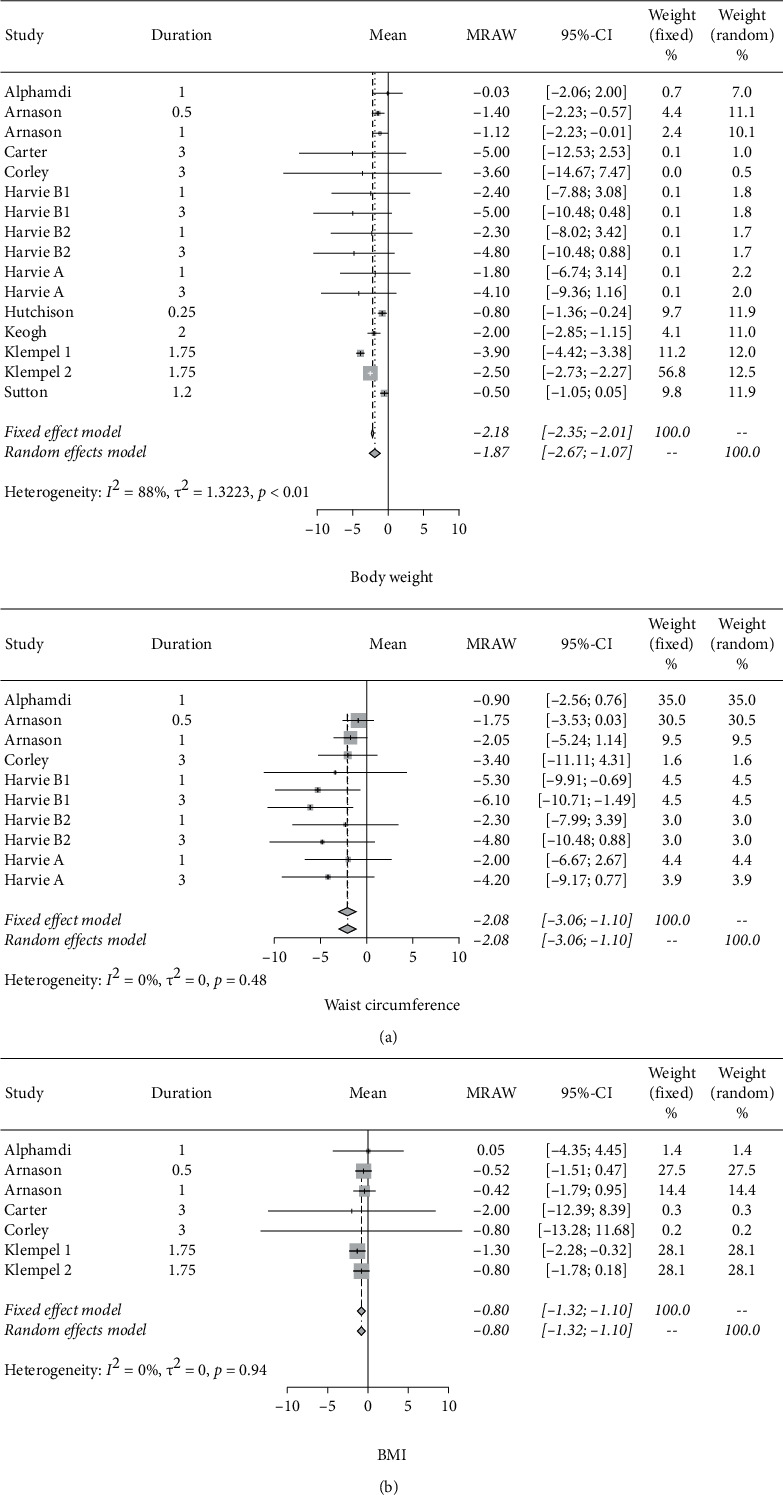
Forest plots for body weight, waist circumference, and BMI.

In addition, the average weight decreased by 2.51 kg (95% CIs: −3.27 to −1.75), whereas the waist circumference reduced by 2.25 cm on average (95% CIs:−3.08 cm to −1.42 cm). BMI reduced by −0.82 kg/m2 (95% CIs: −1.34 to −0.30), as shown in Figure 3.

**Figure 4:**
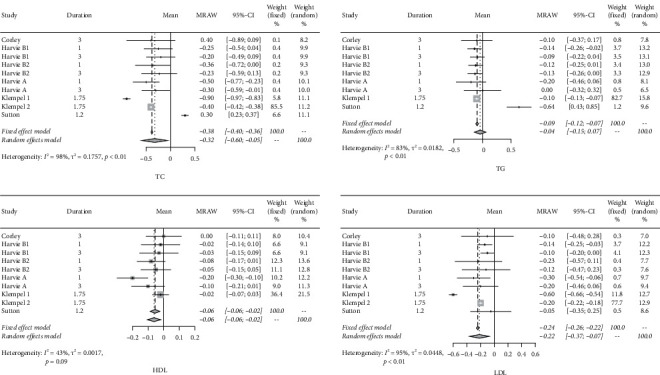
Forest plots for TC, TG, LDL, and HDL.

About lipid metabolism, the forest plots in Figure 4 show that after IF, the triglyceride level decreased, with a mean difference of −0.09 mmol/L and the corresponding 95% CIs of −0.12 to -0.07, whereas the total cholesterol level decreased by 0.38 mmol/L (95% CIs: −040; −0.36), LDL level decreased by 0.22 mmol/L (95% CIs: −0.37; −0.07), and HDL level decreased by 0.06 mmol/L (95% CIs: −0.09; −0.02).

**Figure 5:**
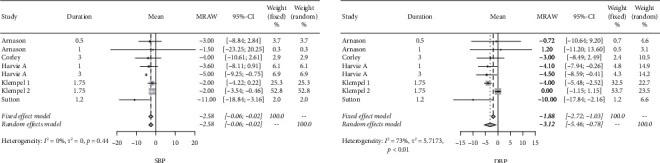
Forest plots for blood pressure.

As far as blood pressure was concerned, systolic blood pressure (SBP) and diastolic blood pressure (DBP) levels dropped on average of −2.58 mmHg (95% CIs: −3.70; −1.46) and −3.12 mmHg (95% CIs: −5.46; −0.78), respectively. The forest plots for these two parameters are presented in Figure 5. Lastly, by leaving one study out in turn, sensitivity analyses were carried out for each outcome, and the results of such analyses indicated the synthesized results are acceptably robust.

## 4. Discussion

The common chronic diseases of MS, such as type 2 diabetes mellitus and cardiovascular disease, are caused by chronic low-grade metabolic inflammation of the body and/or the specific target tissues [20]. The crucial mechanism of metabolic inflammation is the increase of macrophage infiltration in adipose tissue and the level of related inflammatory cytokines, which is also the main cause of insulin resistance. Therefore, to improve and prevent the occurrence and progression of MS, the goal of reducing body weight and fat and improving insulin resistance must be achieved to expect the long-term stable improvement of glycolipid metabolism and prevention of chronic diseases.

We collated a clinical research on the relationship between IF and impaired glucose and lipid metabolism conducted over the past 8 years. This was used to investigate whether IF can improve glycolipid metabolism in patients with impaired glucose and lipid metabolisms. To date, there have been few studies that have focused on the effects of IF diets on impaired glucose and lipid metabolism. The distribution of alternative clinical trials was uneven, ranging from 2 weeks to 2 years, but the main research was concentrated within 3 months. The length of the data was 3 months as the time intercept point for analyzing the efficacy. In all studies, although the IF intervention did not strictly limit the calorie intake, the fasting blood glucose was reduced by an average of 0.15 mmol/L (95% CIs: −0.23; −0.06). HbA1c also decreased by 0.08% (95%CIs:−0.25; −0.10). In terms of the decrease of blood glucose and HbA1c, the improvement effect of IF on glucose metabolism is not significant, since part of the population in the study were patients with impaired glucose and lipid metabolism but not patients with previous glucose abnormalities. Only Arnason and Corley's study [14, 16] included patients with glucose abnormalities and contributed 3.2% of the population. Alghamdi, Corley, and Carter [11, 15, 16] considered abnormal HbA1c and their studies contributed only 42.8%. As expected, no substantial changes in fasting blood glucose after intervention were observed since the proportion of subjects with glucose abnormality was relatively small. Furthermore, the IF diet is different from a ketogenic or low-calorie diet, which does not strictly limit carbohydrate intake; hence, the direct impact on blood glucose in the short term was not obvious. However, IF is certainly beneficial for fasting blood glucose control [21].

Insulin sensitization and improvement of insulin resistance are not only international research hotspots but also important strategies in the current treatment of type 2 diabetes and cardiovascular diseases [22, 23]. Central obesity and increased visceral fat mass are the main risk factors for chronic diseases. A follow-up study of over 15 years with the US men and women demonstrated that for every 1 kg increase in men, the risk of type 2 diabetes increased by 2 times, but the risk increased 7 times in women [24, 25]. Lyall et al. [25] concluded through a study of over 110,000 subjects that the risk of hypertension and atherosclerotic coronary heart disease (CHD) increased by 64% and 35%, respectively, for each 4.83 kg/m^2^ increase in BMI. In addition, adipose tissue inflammation is a key factor causing insulin resistance [26], besides adipocyte differentiation and adipocyte hypertrophy lead to a vicious circle of inflammation [27]. Therefore, reduction in body weight or visceral fat implied the improvement in insulin sensitivity, thereby preventing diabetes and cardiovascular disease. All the included studies showed that IF intervention resulted in weight loss with an average decrease in BMI of 0.8 kg/m^2^ (95% CIs:−1.32; −0.28), an average decrease in weight of 1.87 kg (95% CIs: −2.67; −1.07), and an average decrease in waist circumference of 2.08 cm (95% CIs: −3.06; −1.10). Harvie reported after 3 months of IF intervention, the bodyweight reduced periodically compared with that of one-month intervention; in specific, the weight loss was 4.1–5.0 kg at 3 months [13]. Corley et al. and Carter et al. [15, 16] also reported similar weight reduction (−3.6 kg and −5.0 kg, respectively), and the waist circumference reduction (3.4 cm and 6.1 cm, respectively) was significant. HOMA-IR reduced by an average of 0.31 (95% CIs: −0.44; −0.19). The decrease in the insulin level reflects the increase in insulin sensitivity. HOMA-IR is the insulin resistance index and is also the gold standard for evaluating insulin sensitivity. Analysis of insulin sensitivity after IF diet intervention showed that insulin levels reduced by an average of 13.25 mU/L (95% CIs: −16.69; −9.82), compared to the clinical studies of the typical oral insulin sensitization pioglitazone [28], in which fasting insulin levels decreased by 7.9 mU/L on average, and 2 h postprandial glucose decreased by 11.2 mU/L after oral intake of pioglitazone for 16 weeks. The scope of insulin reduction after IF intervention was similar to oral insulin sensitization. Although insulin sensitizations reduce insulin levels and improve insulin sensitivity, the side effects of the medication, such as weight gain, are inevitable, which will affect the long-term effects of patients with impaired glucose and lipid metabolism. However, dietary intervention alone achieved the ideal effect of improving insulin sensitivity [29]. This type of adjuvant therapy to improve lifestyle is worth recommending. Although blood glucose and HbA1c have not been significantly improved within 3 months, long-term improved glucose control can be expected with the significant improvement in insulin sensitivity [30].

In terms of lipid metabolism, IF diet intervention effectively reduced plasma total cholesterol and LDL [31]. The total cholesterol level reduced by an average of 0.32 mmol/L (95% CIs: −0.60; −0.05), LDL reduced by an average of 0.22 mmol/L(95% CIs: −0.37; −0.07), and triglyceride level decreased by 0.04 mmol/L (95% CIs: −0.15; −0.07), and compared with the studies by Yancy et al. [32] and McDonald and Cervenka [33] on the ketogenic diet with targeted treatment effects for diabetes, although, the intervention duration was close (16 weeks and 10 weeks, respectively), total cholesterol only reduced by 0.07 mmol/L and 0.16 mmol/L, respectively, on average. Thus, an IF diet may have similar effects on improving lipid metabolism to a ketogenic diet. The IF diet intervention not only showed effects on weight loss, insulin sensitivity, and glycolipid metabolism but it may also lower blood pressure [34]. The average systolic blood pressure was reduced by 3.12 mmHg (95% CIs: −5.46; −0.78), and the average diastolic blood pressure was reduced by 2.58 mmHg (95% CIs−3.70; −1.46).

Furthermore, our findings regarding diabetes mellitus are consistent with those of a previous meta-analysis conducted by Wang et al. [35]; however, the key to diabetes prevention lies in the regulation of MS-related biomarkers. Only two of the studies in our meta-analysis focused on MS, and neither of these provided clear results. The present study fills this gap in the literature by investigating the effects of IF diets on impaired glucose and lipid metabolism. We also present suggestions for the improvement of indicator selection when choosing biomarkers with which to measure glycolipid metabolism. It can take some time before impaired glucose and lipid metabolism progress to serious illness, and this might cause complacency in some people with impaired glycolipid metabolism, neglecting to address and correct the problem. However, treatment of the resultant chronic diseases places a heavy economic burden on individuals and society [36]. The negative effects on the quality of life of extremely low-calorie diets cause most to fail as such self-deprivation cannot be sustained for long periods. Therefore, weight-reducing diets that can feasibly be maintained on a long-term basis without detriment to the quality of life are much needed, both by the overweight and obese as well as by those with impaired glucose and lipid metabolism.

### 4.1. Limitations

In total, ten studies utilizing twelve dietary interventions were included in the current meta-analysis. The types of IF used in the trials varied, as did the ethnicities of participants. Thus, it was not possible to group studies by ethnicity and/or types of IF. Similarly, among the studies included, only two (incorporating four types of dietary interventions between them) measured participant insulin levels, only three (with a total of five types of dietary interventions) measured HbA1c, and only three (with a total of eight types of dietary interventions) measured HOMA-IR. This made it impossible to group the studies by types of biomarkers. More studies are required to allow more stratified comparisons based on disease, ethnicity, type of intervention, and duration of intervention.

However, despite these limitations, our results suggest that IF diets have therapeutic effects on individuals with impaired glucose and lipid metabolism, regardless of the specific type of IF intervention or the particular clinical manifestations of the impaired glucose and lipid metabolism. In addition, as there are no calorie restrictions required in IF diets, they do not have negative effects on the quality of life as exhibited by very-low-calorie and very-low-carbohydrate diets. Thus, diets based on IF are executable and sustainable. As such, they are a valuable adjunct to the clinical treatment of patients with impaired glucose and lipid metabolism.

## 5. Conclusion

The current study provided some support on the fact that the IF diet is an effective therapeutic option for patients with impaired glucose and lipid metabolism, and it may improve glucose and lipid metabolism, as well as achieve significant weight loss and improve insulin resistance.

## Figures and Tables

**Table 1:** Summary of participants' characteristics of studies included.

	Study	Country	Study subdivision	*N*	Follow up	Dietary intervention	Age	BMI (kg/m^2^)	Population
1	Sutton et al. [9]	USA		8	5 weeks	Early time-restricted feeding (eTRF); no calorie restriction	56 ± 9	32.2 ± 4.4	Overweight/obese with prediabetes
2	Alphamdi et al. [11]	Saudi Arabia		36	44 days	Ramadan; no calorie restriction	49.50 ± 11.91	32.89 ± 5.47	Overweight/obese with diabetes
3	Harvie et al. [13]	UK		53	6 months	Intermittent energy restriction (IER); 75% calorie restriction on two consecutive days each week	40.1 ± 4.1	30.7 ± 5	Non-diabetic obese/overweight
4	Harvie et al. [12]	UK	*B*1	37	4 months	Intermittent energy and carbohydrate restriction (IECR); (70% energy restriction and 40 g carbohydrate)	45.6 ± 8.3	29.6 ± 4.1	Non-diabetic obese/overweight
			*B*2	38		Intermittent energy and carbohydrate restriction (IECR) + protein and fat (PF); (70% energy restriction and 40 g carbohydrate) + unlimited lean meat, fish, eggs, tofu, MUFA and PUFA on restricted days	48.6 ± 7.3	31.0 ± 5.7	Non-diabetic obese/overweight
5	Arnason et al. [14]	Canada		10	6 weeks	Intermittent fasting (IF)	53.8 ± 9.11	36.9 ± 8.29	Diabetes
6	Corley et al. [16]	New Zealand		19	12 weeks	Diet with non-consecutive fasting days (fasting 500 kcal for women, 600 kcal for men)	58 (42–74)^1^	36.8 ± 5.2	Obese with diabetes
7	Carter et al. [15]	Australia		70	12 months	Intermittent energy restriction (IER); 500–600 kcal for 2 days	61 ± 9	35 ± 5.8	Diabetes
8	Hutchison et al. [17]	Australia		15	7 days	9-hour time-restricted feeding (TRF)	55 ± 3	33.9 ± 0.8	Overweight or obesity
9	Keogh et al. [18]	Australia		19	12 months	Intermittent energy restriction (IER); 1300 kcal for 1 week, normal diet for 1 week	59.5 ± 8.7	33.8 ± 3.1	Non-diabetic obese/overweight
10	Klempel et al. [19]	USA	1	28	10 weeks	Intermittent fasting calorie restriction-liquid diet (IFCR-L); 30% restricted calorie	47 ± 2	35 ± 1	Non-diabetic obese
			2	26		Intermittent fasting calorie restriction-food based diet (IFCR-F); 30% restricted calorie	48 ± 2	35 ± 1	Non-diabetic obese

^*∗*^ *N* represents number of participants recruited in the study; ^*∗*^*B*1 and *B*2 represent difference types of intermittent fasting dietary intervention included in the same study; ^*∗*^1 and 2 represent different types of dietary intervention included in the same study, the superscript number 1 represents age in median with a range from 42–74 years old.
